# Cardiovascular implications and physical activity in middle-aged and older adults with a history of COVID-19 (CV COVID): a protocol for a randomised controlled trial

**DOI:** 10.1186/s13063-023-07360-7

**Published:** 2023-05-13

**Authors:** Mushidur Rahman, Sophie L. Russell, Nduka C. Okwose, Olivia M. A. Hood, Amy E. Harwood, Gordon McGregor, Stuart M. Raleigh, Hardip Sandhu, Laura C. Roden, Helen Maddock, Prithwish Banerjee, Djordje G. Jakovljevic

**Affiliations:** 1 Research Centre for Health and Life Sciences, Institute for Health and Wellbeing, Faculty of Health and Life Sciences, Coventry University, Alison Gingell Building, 20 White Friars Street, Coventry, CV1 2DS UK; 2 Department of Cardiology, University Hospitals Coventry and Warwickshire NHS Trust, Coventry, UK; 3 School of Biosciences, College of Biomedical and Life Sciences, Cardiff University, Cardiff, UK; 4 Warwick Clinical Trials Unit, Warwick Medical School, University of Warwick, Coventry, UK; 5 Health Through Physical Activity Lifestyle and Sport Research Centre & Division of Exercise Science and Sports Medicine, Department of Human Biology, Faculty of Health Sciences, University of Cape Town, Cape Town, South Africa

**Keywords:** COVID-19, Coronavirus, Cardiovascular system, Randomised controlled trial, Echocardiography, Arterial stiffness, Physical activity

## Abstract

**Background:**

The clinical manifestation of COVID-19 is associated with infection and inflammation of the lungs, but there is evidence to suggest that COVID-19 may also affect the structure and function of the cardiovascular system. At present, it is not fully understood to what extent COVID-19 impacts cardiovascular function in the short- and long-term following infection. The aim of the present study is twofold: (i) to define the effect of COVID-19 on cardiovascular function (i.e. arterial stiffness, cardiac systolic and diastolic function) in otherwise healthy individuals and (ii) to evaluate the effect of a home-based physical activity intervention on cardiovascular function in people with a history of COVID-19.

**Methods:**

This prospective, single-centre, observational study will recruit 120 COVID-19-vaccinated adult participants aged between 50 and 85 years, i.e. 80 with a history of COVID-19 and 40 healthy controls without a history of COVID-19. All participants will undergo baseline assessments including 12-lead electrocardiography, heart rate variability, arterial stiffness, rest and stress echocardiography with speckle tracking imaging, spirometry, maximal cardiopulmonary exercise testing, 7-day physical activity and sleep measures and quality of life questionnaires. Blood samples will be collected to assess the microRNA expression profiles, cardiac and inflammatory biomarkers, i.e. cardiac troponin T; N-terminal pro B-type natriuretic peptide; tumour necrosis factor alpha; interleukins 1, 6 and 10; C-reactive protein; d-dimer; and vascular endothelial growth factors. Following baseline assessments, COVID-19 participants will be randomised 1:1 into a 12-week home-based physical activity intervention aiming to increase their daily number of steps by 2000 from baseline. The primary outcome is change in left ventricular global longitudinal strain. Secondary outcomes are arterial stiffness, systolic and diastolic function of the heart, functional capacity, lung function, sleep measures, quality of life and well-being (depression, anxiety, stress and sleep efficiency).

**Discussion:**

The study will provide insights into the cardiovascular implications of COVID-19 and their malleability with a home-based physical activity intervention.

**Trial registration:**

ClinicalTrials.gov NCT05492552. Registered on 7 April 2022.

**Supplementary Information:**

The online version contains supplementary material available at 10.1186/s13063-023-07360-7.

## Introduction

Severe acute respiratory syndrome coronavirus 2 (SARS-CoV-2) caused the coronavirus disease outbreak (COVID-19) in 2019 [1]. The virus spread worldwide and was declared a pandemic by the World Health Organization (WHO) [2]. COVID-19 presents as an acute respiratory infection with symptoms ranging from mild to severe [3]. This highly infectious disease has claimed an estimated six million deaths worldwide [4]. COVID-19 transmission occurs directly through respiratory droplets (aerosol and droplet) and indirectly through faecal-oral routes.

Whilst the virus primarily affects the respiratory system, there has been evidence to suggest effects on other organ systems including the cardiovascular system [5]. Pre-existing conditions such as hypertension, cardiovascular disease (CVD), obesity, diabetes, renal disease, chronic respiratory disease, tumours or cancers are associated with higher severity and increased fatality of COVID-19 infection [6].

Many reports have highlighted that COVID-19 with cardiovascular involvement leads to poor prognosis and outcomes [7, 8]. A fivefold increase in the death rate in those with pre-existing CVD was reported [6]. A previous study found myocardial injury was associated with the increased fatality of COVID-19, with inflammation being a potential mechanism for myocardial injury [9]. Limited number of studies reported the cardiovascular effects of COVID-19 in previously healthy individuals.

To maintain overall health, physical activity has been beneficial to accelerate recovery in chronic conditions [10]. The importance of exercise has been highlighted in many studies for the prevention and rehabilitation of cardiovascular function. Liu et al. outlined that rehabilitation may cause respiratory muscle strengthening and improvement of respiratory system function [11]. Physical activity can improve health in low-grade inflammation (associated with ageing) and COVID-19 [12–16]. The WHO highlighted that home-based physical activity provides a safe, cheap and controllable intervention for all ages affected by COVID-19 [17]. Home-based physical activity has been shown to reduce blood pressure [18] and improve quality of life (QoL) [19]. Given the novelty of this virus, there is a lack of research on physical rehabilitation post-COVID-19 infection. As some individuals presented with COVID-19 pneumonia, home-based exercise in the elderly improved cardiac peak flow and physical function which prevented aspiration pneumonia [20]. Tang et al. found that a home-based exercise programme improved the functional capacity and overall quality of life in those discharged after being admitted with COVID-19 [21]. A case report showed exercise rehabilitation improved the 6-min walk test and stress test results where the patient had oxygen (O_2_) desaturation in the initial testing [22]. Similarly, Stavrou et al. demonstrated that 8 weeks of an unsupervised rehabilitation program can improve many symptoms of long COVID-19 syndrome [23]. Previous studies have also found that a 6-month home-based cardiac rehabilitation for breast cancer patients was safe, improved peak O_2_ consumption and overall physical fitness and is an effective method to encourage participation in physical activity [24]. Improvement in QoL, exercise tolerance and haemodynamic function was also reported following a home-based physical activity intervention in middle-aged to older individuals with heart failure [25]. More recently, studies have shown that home-based physical activity intervention improves exercise capacity and enhances recovery and QoL in COVID-19 survivors [26, 27].

Cardiovascular involvement in healthy individuals with a history of COVID-19 infection is still uncharacterised. It is important to investigate the initial signs of changes in cardiovascular structure and function to improve understanding of the effects of this novel disease. This can inform health care services by providing evidence for the need for comprehensive cardiovascular screening and early interventions to prevent deterioration if cardiovascular function in people with a history of COVID-19.

The present study is designed to evaluate the effects of COVID-19 on cardiovascular structure and function and to determine their amelioration with physical activity. The aim of the present study is twofold: (i) to define the effect of COVID-19 on cardiovascular function (i.e. arterial stiffness, cardiac systolic and diastolic function) in otherwise healthy individuals and (ii) to evaluate the effect of a home-based physical activity intervention on cardiovascular function in people with a history of COVID-19. We will test the following two hypotheses: (i) cardiovascular function will be significantly reduced in people with a history of COVID-19, and (ii) a home-based physical activity intervention will significantly improve cardiovascular function and quality of life in people with a history of COVID-19.

## Methods

### Study design, setting and ethics

The present single-centre, prospective, observational and interventional, randomised controlled study will be undertaken at the Coventry University Cardiovascular Research Laboratory between January 2022 and August 2023.

**Fig. 1:**
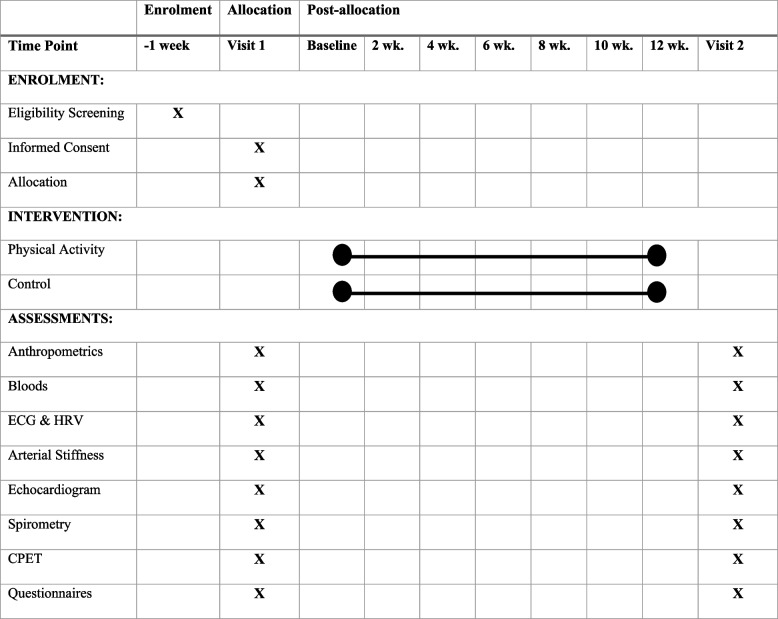
Study timeline: time schedule of enrolment, intervention and assessment for visits 1 and 2. ECG, electrocardiogram; CPEX, Cardiopulmonary exercise stress test; HRV, heart rate variability; wk., week

Ethical approval was granted by the Coventry University Research Ethics Committee (P125303) and the UK Health Research Authority National Health Service East Midlands – Leicester South Research Ethics Committee (22/EM/0090). The study will be conducted in accordance with the Declaration of Helsinki. The study was prospectively registered on ClinicalTrials.gov (NCT05492552) on 7 April 2022. The participant timeline is shown in Fig. 1.

### Sample size

**Fig. 2:**
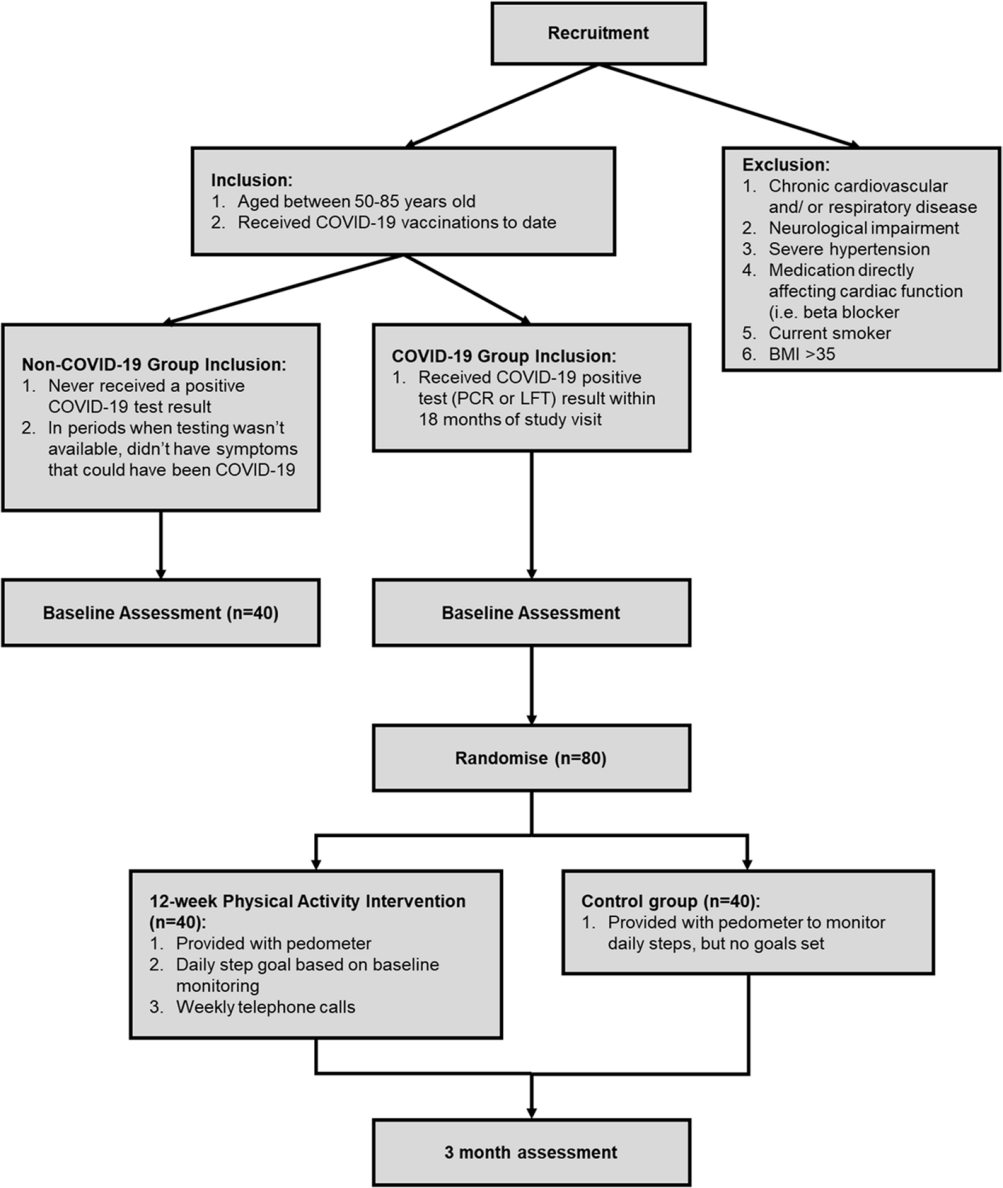
Study design, inclusion and exclusion criteria

One hundred twenty participants aged between 50 and 85 years will be recruited for the study. This will include 80 participants with a history of COVID-19 and 40 healthy individuals without a history of COVID-19 to complete baseline assessments. Additionally, participants with a history of COVID-19 will be randomly assigned (1:1) to either a 12-week home-based physical activity intervention or usual care control group. The study design is outlined in Fig. 2. The primary outcome measure is LV GLS, as a measure of cardiac systolic function. With a type I error of 0.05 and an intended high power of > 90%, 40 participants per group are required to detect a 3% difference in LV GLS between the COVID-19 and non-COVID groups. In response to the physical activity intervention within the COVID-19 group, assuming a standard deviation of 2.5% and an estimated attrition rate of ~ 20% in the intervention period of the study, 40 participants per group will be required, providing the overall recruitment target of 80 participants with a history of COVID-19.

### Randomisation

Randomisation numbers will be generated by secure web-based randomisation to maintain concealment and minimise selection bias. The randomisation process will be performed by the researchers, and the principal investigator will generate the randomisation numbers. A randomisation sequence was computer generated, with each allocation assigned a number. Following baseline testing, each participant was asked to pick out a number from an opaque box. Participants were blinded to their allocation to ensure a ‘usual’ baseline week was obtained; however, due to the nature of the intervention, researchers (i.e. outcome assessors and analysts) were unblinded to the allocation.

### Recruitment

Participants will be recruited through email invitations sent to Coventry University Faculties and social media (Twitter, Facebook). Professional social media accounts will be created according to the British Psychological Society guidelines [28]. Recruitment from NHS facilities (primary and secondary care) will require the clinical care team to screen potential participants, and if participants consent to be contacted, the researchers will contact the participant.

### Inclusion and exclusion criteria

Individuals with a history of COVID-19 diagnosed clinically via polymerase chain reaction (PCR) or lateral flow test (LFT) will be recruited into the COVID-19 group whilst those who have never received a positive COVID-19 diagnosis will be recruited into the non-COVID-19 group. The full list of the inclusion and exclusion criteria can be seen in Table 1.

**Table 1:** Study inclusion and exclusion criteria

Inclusion criteria	Exclusion criteria
i) Aged 50–85 years ii) COVID-19 vaccinated, i.e. at least two doses of the NHS-approved vaccine iii) *COVID group:* PCR- or LFT-confirmed COVID-19 diagnosis between 28 days post-infection and < 18 months from initial visit iv) *Non-COVID group:* never received a positive COVID-19 diagnostic test using PCR or LFT and during periods when testing was not available v) Did not have symptoms that could have been COVID-19	i) Known chronic respiratory or cardiovascular conditions, i.e. COPD, emphysema, pulmonary hypertension, coronary artery disease ii) Severe hypertension (systolic blood pressure > 180 mmHg, diastolic blood pressure > 120 mmHg) iii) Acute or chronic neurological impairment or progressive neurological disease iv) Use of medication known to directly affect cardiac function (i.e. beta-blockers) v) Current smoker vi) Body mass index > 35 kg/m^2^ vii) Previously ventilated during COVID-19 infection viii) Exceeding current physical activity guidelines defined by the WHO

*Abbreviations*: *COVID-19* Coronavirus 2019, *NHS* National Health Service, *PCR* polymerase chain reaction, *LFT* Lateral flow test, *COPD* Chronic obstructive pulmonary disease, WHO World Health Organization

### Study assessments

During the first visit, participants will undergo screening to ensure they meet the study inclusion/exclusion criteria. Participants will be given an opportunity to ask questions to ensure they understand all procedures of the study, and a written informed consent will be taken afterwards. Participants will be informed that they are able to withdraw from the study at any point. If a participant becomes ineligible during the study period, they will be withdrawn from the study.

#### Blood sample

Blood samples will be collected by a phlebotomy-trained member of the research team according to Good Clinical Practice (GCP). Blood samples will be analysed for cardiac and inflammatory biomarkers: cardiac troponin T (cTnT); N-terminal pro B-type natriuretic peptide (NT-proBNP); tumour necrosis factor alpha (TNFα); interleukins 1β, 6 and 10; C-reactive protein (CRP), d-dimer and vascular endothelial growth factors (VEGFs). In addition, microRNA expression profiles will be assessed (TaqMan™ Array Human MicroRNA A Cards, Applied Biosystems, USA).

#### Cardiorespiratory fitness

A medical history, physical examination and 12-lead electrocardiogram coupled with heart rate variability will be performed prior to a maximal cardiopulmonary exercise test. The test will include a ramped exercise protocol using an electronically braked semi-recumbent cycle ergometer (Ergoselect 600, Germany) and gas exchange monitoring (Ultima Series, Medgraphics, UK) to measure peak oxygen consumption (VO_2peak_) and lung volumes. A maximal ramp test will be undertaken with the workload starting at 0 W. Pedal frequency will be maintained at 60–70 revolutions per minute. Participants will be verbally motivated during the tests according to an agreed template to ensure uniformity across all tests. Participants perceived exertion will be monitored using the BORG scale. The test will be terminated if participants’ reach peak exertion (i.e. RER > 1.1), the participant requests to stop before the exercise protocol completion or any indication for exercise termination described in the ATS/ACCP statement on cardiopulmonary exercise testing [29].

#### Cardiovascular function

Cardiac and vascular parameters will be assessed at rest and peak cardiopulmonary exercise using non-invasive methods (Starling SV – Baxter Healthcare Ltd., USA) and echocardiogram. An echocardiogram will be performed using ultrasound to acquire 2D real-time images of the heart to assess cardiac structure and function (Vivid IQ, GE Healthcare, USA). Participants will be asked to undress to the waist, and gowns will be offered to all participants. An ultrasound probe will be placed on the chest with lubricating gel to form good contact between the probe and skin for image acquisition. Echocardiography will be performed at rest and at peak exercise.

Arterial stiffness, a measure of arterial function, will be assessed at rest using non-invasive gold standard techniques which allows for both pulse wave analysis (PWA) and pulse wave velocity (PWV)measurements (Sphygmacor device; AtCor Medical, NSW, Australia). A blood pressure cuff will be placed on the participants’ upper left arm for PWA assessment. For PWV assessment, a tonometer will be placed against the neck and a blood pressure cuff will be placed on the upper thigh to simultaneously measure carotid artery pulse wave velocity and femoral artery blood pressure.

#### Questionnaires

Participants will be asked to complete four questionnaires to assess quality of life, fatigue, depression, anxiety, stress and sleep quality during both visits. Questionnaires include Short Form 36 (SF-36); Chalder Fatigue Scale; Depression, Anxiety and Stress Scale-21 (DASS-21); and Pittsburgh Sleep Quality Index (PSQI). Questionnaires will be checked to ensure completeness of data.

#### Step count, daily activity and sleep

Participants will be provided with a pedometer (Omron Walking Style IV, Japan) to record the daily step count throughout the duration of the study. Step count logs will be completed with daily steps recorded. Physical activity and sleep will be measured over 7 days using an accelerometer worn on the wrist (ActTrust and ActTrust 2, Condor Instruments, Brazil) during the baseline week and the week preceding the end of the intervention.

### Intervention

#### Physical activity intervention group

The exercise intervention group will undergo a 12-week home-based physical activity intervention. The participants will be asked to increase their daily steps by at least 2000 steps per day from baseline, e.g. walking for approximately 30 min in addition to baseline, 5 days a week. A large cohort study of 9306 participants demonstrated that an incremental increase in physical activity of 2000 steps per day reduced the risk of cardiovascular events by 10% [30]. A pedometer will be provided to all participants with a diary sheet to record the number of steps per day. Weekly telephone calls lasting 10–15 min each will be made to each participant in the intervention group to provide an opportunity for the participants to ask any questions and for the research staff to encourage the participant to achieve their step goal. Suitable days and times will be arranged for phone calls to take place. After the intervention period, i.e. 13 weeks from the date of the initial visit, participants will be invited to visit the laboratory for follow-up assessments. One week will be allowed for the participants to record their baseline step count. On the first day of the second week, researchers will contact the participant to inform them of which group they have been allocated to. The same outcomes will be measured during the follow-up visit to determine a value of change.

#### Control group

The control group will be provided with a pedometer to record daily step count but will not need to increase daily step count. Once the 12 weeks have been completed, participants will be invited to attend the research laboratory for follow-up assessments.

### Outcome measures

The primary outcome measure is mean change in left ventricular global longitudinal strain after 12 weeks of the study intervention. Secondary outcomes are mean changes from baseline to the end of the intervention period in arterial stiffness (pulse wave velocity and augmentation index), measures of systolic and diastolic function of the heart (i.e. left ventricular ejection fraction, cardiac output, stroke volume, cardiac power, early-to-late filing ratio, right ventricular global longitudinal strain, left atrial strain, ventricular-arterial coupling, right ventricular diastolic function), heart rate variability, functional capacity, lung function, inflammatory and cardiac biomarkers, quality of life and well-being (depression, anxiety, stress, fatigue and sleep measures). Sleep measures include bedtime (h:min), wake-up time (h:min), time-in-bed (h), total sleep time (h), sleep onset latency (min), sleep efficiency (%), number of awakenings and wake after sleep onset (WASO, min), number and timing of naps, inter-daily variability and stability (as markers of circadian rhythmicity), and timing and nature of light exposure during a 24-h period. Additionally, we will assess the differences in the COVID-19 group vs. the non-COVID-19 group.

### Data collection storage and monitoring

Data will be collected by the research team members, and only individuals authorised by the chief investigator will have access to the data. All data will be processed in accordance with the General Data Protection Regulation 2016 and the Data Protection Act 2018. Data will be given a unique participant identification number. Data will be documented in paper-based case report form (CRF), all data will be uploaded to an access-controlled OneDrive folder from a password-protected computer and all paper documents will be stored in a locked cabinet. Data entry will be checked by two of the research team members to minimise errors. Data will be archived in accordance with institutional guidelines after study closure and will be stored up to 5 years. No formal data monitoring committees have been organised for this study. Research team members, led by the chief investigator, will monitor the study throughout the period of the research study.

### Data analysis methods

Initially, intention-to-treat analysis will be used, and all data will be used from all participants according to the groups to which they have been assigned to. Secondary analysis will involve per-protocol analysis, which will analyse the adherence to the intervention. All obtained data will be used for the analysis. To minimise missing data, participants will be contacted weekly by the research team if in the intervention group and call to all control participants for their follow-up visit as a reminder. As the study requires a single follow-up visit, only complete cases will be analysed, and no data imputation will be used. All statistical analyses will be carried out using the IBM SPSS software, version 28. Descriptive statistics will be presented as means and standard deviations (SD). All data will be assessed for normality using the Kolmogorov–Smirnov test. Independent samples *T*-test or Mann–Whitney *U*-test, as appropriate, will be used to assess the differences in variables between COVID and non-COVID groups. Paired-samples *T*-test or Wilcoxon signed-rank test, as appropriate, will be used to evaluate the differences in variables pre- and post-intervention. Pearson’s or Spearman’s tests (as appropriate) will be used to determine correlation coefficient (*r*). A *p*-value of < 0.05 will be considered statistically significant.

### Dissemination policy

Once the study has been completed, and all data will be analysed and published in peer-reviewed journals and presented at appropriate conferences. Participants will have an opportunity to receive a copy of the scientific publication(s) with the complete, unidentifiable dataset.

## Discussion

Short- and long-term effects of COVID-19 on the cardiovascular structure and function in otherwise healthy, middle-aged and older people are still not fully investigated. Studies have shown that although COVID-19 is viral respiratory pneumonitis, diverse cardiovascular manifestations have been seen such as myocardial injury, arrhythmias, acute coronary syndrome, heart failure and thromboembolism in individuals with pre-existing medical conditions [31]. It was suggested that those individuals who did not initially present with the most common symptom of COVID-19 (cough) had cardiac symptoms as the first manifestation of disease [31]. Furthermore, it has been demonstrated that myocardial injury in those infected with COVID-19 was independently associated with increased mortality rates [31].

Multiple studies have evidenced myocardial injury in COVID-19 patients by detecting elevated cardiac biomarkers, abnormal ECG and echocardiogram findings [32, 33]. Patients who presented as asymptomatic or had mild symptoms went on to develop persistent symptoms [34]. Those individuals who had only mild symptoms of COVID-19 had evidence of cardiac injury in cardiac magnetic resonance imaging (MRI) [5].

The present study will investigate the effects of COVID-19 in otherwise healthy, middle-aged and older people. Although many reports from the early phases of COVID-19 demonstrated cardiovascular manifestation of this novel virus, these studies have been conducted in predominantly hospitalised patients with pre-existing medical conditions. The present study will test the hypothesis that patients with history of COVID-19 will demonstrate post-COVID cardiovascular (clinical or subclinical) implications, and these will be ameliorated with a home-based physical activity intervention.

## Supplementary Information

**Additional file 1: Table 1.** Clinical trials registration information.

## Data Availability

The data collected during this trial will be processed electronically. The entirety of the final trial dataset will be shared by the investigators upon completion of the study. The principal investigator and all researchers involved in this trial will have access to the final trial dataset. Participants will be notified of their individual findings as requested.

## Abbreviations

BMI: Body mass index
CPET: Cardiopulmonary exercise test
CI: Cardiac index
COVID-19: Coronavirus 2019
CO: Cardiac output
cTnT: Cardiac troponin T
CRP: C-reactive protein
DASS-21: Depression Anxiety Stress Scale-21
ECG: Electrocardiogram
GCP: Good Clinical Practice
HRV: Heart rate variability
IL1 beta: Interleukin-1 beta
IL6: Interleukin-6
IL10: Interleukin 10
LFT: Lateral flow test
NT-proBNP: N-terminal pro B-type natriuretic peptide
PCR: Polymerase chain reaction
PSQI: Pittsburgh Sleep Quality Index
QoL: Quality of life
SARS-CoV-2: Severe acute respiratory syndrome coronavirus 2
SD: Standard deviation
SF-36: Short Form-36
SV: Stroke volume
SVI: Stroke volume index
VEGFs: Vascular endothelial growth factors
WHO: World Health Organization
