# Supplementary material for: Cardiovascular implications and physical activity in middle-aged and older adults with a history of COVID-19 (CV COVID): a protocol for a randomised controlled trial
Source: Trials. 2023 May 13;24:328. doi: 10.1186/s13063-023-07360-7 (PMC10181919; doi:10.1186/s13063-023-07360-7)
Supplement: Supplementary file 1 — Additional file 1: Table 1. Clinical trials registration information. [file 13063_2023_7360_MOESM1_ESM.docx]

**Supplementary Data:**

Table 1 – Clinical Trials Registration information

| **Data category** | **Information** |
| --- | --- |
| **Primary registry and trial identifying number** | ClinicalTrials.gov NCT01143272 |
| **Date of registration in primary registry** | 11 June, 2010 |
| **Secondary identifying numbers** | N/A |
| **Source(s) of monetary or material support** | Coventry University, UK |
| **Primary sponsor** | Coventry University, UK |
| **Secondary sponsor(s)** | N/A |
| **Contact for public queries** | Email: [cvresearch@coventry.ac.uk](mailto:cvresearch@coventry.ac.uk) |
| **Contact for scientific queries** | Email: [Djordje.jakovljevic@coventry.ac.uk](mailto:Djordje.jakovljevic@coventry.ac.uk) |
| **Public title** | Cardiovascular Function and Physical Activity in COVID-19 (CV-COVID) |
| **Scientific title** | Cardiovascular implications and physical activity in middle-age and older adults with a history of COVID-19 (CV COVID) |
| **Countries of recruitment** | United Kingdom |
| **Health condition(s) or problem(s) studied** | COVID-19, Cardiovascular disease |
| **Intervention(s)** | Physical Activity: Increase daily step count by 2,000 from baseline coupled with participant support via weekly telephone calls. |
| **Key inclusion and exclusion criteria** | Ages Eligible for Study: 50 Years to 85 Years (Adult, Older Adult)  Sexes Eligible for Study: All  Accepts Healthy Volunteers: Yes  Inclusion Criteria:   - Between 50-85 years old - Up to date with all COVID-19 vaccinations - COVID participants - had a positive test for COVID-19 over 28 days of initial visit but before 18 months. Non-COVID participants - never received a positive COVID-19 test result, and no symptoms during periods where testing was unavailable.   Exclusion Criteria:   - Chronic respiratory and cardiovascular conditions i.e., chronic obstructive pulmonary disease (COPD), emphysema, pulmonary hypertension, coronary artery disease - Severe hypertension - Acute or chronic neurological impairment or progressive neurological disease - Use of medication known to directly affect cardiac function - Current smoker - Body mass index > 35 kg/m2 - People who exceed current physical activity guidelines defined by the World Health Organization (WHO). |
| **Study type** | Intervention Allocation: randomized Intervention model: parallel assignment Masking: Open label Primary purpose: Treatment |
| **Date of first enrolment** | April 7, 2022 |
| **Target sample size** | 120 |
| **Recruitment status** | Recruiting |
| **Primary outcome(s)** | Difference and change in left ventricular global longitudinal strain.   1. Difference in left ventricular global longitudinal strain (LV-GLS) between COVID and non-COVID participants at rest and during peak **exercise** on baseline **assessment**. 2. Change in LV-GLS between COVID intervention and COVID usual care group at rest and during peak **exercise** on baseline and end of study **assessments**. |
| **Key secondary outcomes** | Differences (baseline) and changes (baseline to 12 weeks) in the following variables:   1. Pulse wave velocity 2. Augmentation index 3. Resting cardiac output 4. Maximal cardiac output 5. Resting heart rate 6. Maximal heart rate 7. Resting stroke volume 8. Maximal stroke volume 9. Resting systemic vascular resistance 10. Maximal systemic vascular resistance 11. Maximal oxygen uptake (VO2 max) 12. Forced Expiratory Volume 1 (FEV1)/ forced vital capacity (FVC) ratio 13. Heart rate (i.e. times the heart beats per minute) will be measured using non-invasive monitoring technology. 14. Quality of life 15. Depression, anxiety and stress 16. Global sleep efficiency 17. Right ventricular global longitudinal strain 18. Left atrial strain 19. Ventricular arterial coupling 20. Right ventricular diastolic function 21. RR- interval 22. RR interval difference (RMSSD) 23. Standard deviation normal RR intervals (SDNN) 24. Low frequency power (LF) 25. High frequency power (HF) 26. Low frequency power (LFnu) 27. High frequency power (HFnu) |
